# Cancer-Induced Cardiac Dysfunction: Mechanisms, Diagnostics, and Emerging Therapeutics in the Era of Onco-Cardiology

**DOI:** 10.3390/cancers17193225

**Published:** 2025-10-03

**Authors:** Sarama Saha, Praveen K. Singh, Partha Roy, Vasa Vemuri, Mariusz Z. Ratajczak, Mahavir Singh, Sham S. Kakar

**Affiliations:** 1 Department of Biochemistry, All India Institute of Medical Sciences, Rishikesh 249203, India; sarama.bchem@aiimsrishikesh.edu.in; 2 Department of Biochemistry, Maharishi Markandeshwar Medical College & Hospital, MMU, Solan 173229, India; 354214@online.mmusolan.org; 3 Department of Biotechnology, Indian Institute of Technology Roorkee, Roorkee 247667, India; partha.roy@bt.iitr.ac.in; 4 Department of Physiology, University of Louisville, Louisville, KY 40202, USA; sreevasa.vemuri@louisville.edu (V.V.); mahavir.singh@louisville.edu (M.S.); 5 Department of Medicine, University of Louisville, Louisville, KY 40202, USA; mariusz.ratajczak@louisville.edu; 6 Brown Cancer Center, University of Louisville, Louisville, KY 40202, USA

**Keywords:** cancer, cachexia, cardiac dysfunction

## Abstract

**Simple Summary:**

Cancer is not confined to the organ or tissue where it originates but rather exerts widespread effects throughout the body. One of the most challenging manifestations is cancer-induced organ dysfunction, particularly cachexia, a complex syndrome that extends beyond the direct toxicities of treatment. Tumors are known to release cytokines, reactive oxygen species (ROS), and neurohormonal signals that trigger systemic inflammation, metabolic stress, and tissue damage. This inflammatory cascade can significantly impact the heart, contributing to myocardial atrophy, fibrosis, and impaired cardiac function. Chemotherapy has long been recognized as a primary driver of cardiovascular toxicity. Therefore, continuous and comprehensive cardiac monitoring is essential throughout cancer treatment. Echocardiography (ECG), especially with strain imaging, remains the frontline tool for assessing cardiac function, while cardiac Magnetic Resonance Imaging (MRI) offers high-resolution insights into structural and functional changes. Current management strategies combine established cardioprotective agents such as ACE inhibitors, beta-blockers, and statins with emerging approaches, including antioxidants and novel targeted therapies. Promising preclinical research into compounds like Withaferin A (WFA) suggests potential for preventing or mitigating imminent cardiac damage. Addressing cancer-induced cachexia and its cardiovascular consequences requires a multidisciplinary approach, supported by ongoing research and personalized treatment strategies.

**Abstract:**

Cancer-induced cardiac dysfunction has become a major clinical challenge as advances in cancer therapies continue to extend patient survival. Once regarded as a secondary concern, cardiotoxicity is now recognized as a leading contributor to morbidity and mortality among cancer patients and survivors. Its pathophysiology is multifactorial, involving systemic inflammation (e.g., TNF-α, IL-6), oxidative stress driven by reactive oxygen species (ROS), neurohormonal imbalances (e.g., angiotensin II, endothelin-1), and metabolic disturbances. These mechanisms collectively promote cardiomyocyte apoptosis, atrophy, mitochondrial dysfunction, and impaired cardiac output. Cardiac complications may arise directly from cancer itself or as adverse effects of oncologic therapies such as anthracyclines, trastuzumab, and immune checkpoint inhibitors. These agents have been linked to heart failure (HF), systolic dysfunction, and cardiac atrophy, often progressing insidiously and underscoring the importance of early detection and careful monitoring. Current preventive and therapeutic strategies include pharmacological interventions such as ACE inhibitors, beta-blockers, statins, dexrazoxane, and endothelin receptor antagonists like atrasentan. Emerging compounds, particularly Withaferin A (WFA), have shown potential through their anti-inflammatory and cardiac protective properties. In addition, antioxidants and lifestyle modifications may provide supplementary cardioprotective benefits, while interventional cardiology procedures are increasingly considered in selected patients. Despite encouraging progress, standardized treatment protocols and robust long-term outcome data remain limited. Given the heterogeneity of cancer types and cardiovascular responses, a personalized and multidisciplinary approach is essential. Continued research and close collaboration between oncologists, cardiologists, and basic scientists will be the key to advancing care, reducing treatment-related morbidity, and ensuring that improvements in cancer survival are matched by preservation of cardiovascular health.

## 1. Introduction

### 1.1. The Global Cancer Burden: Incidence, Mortality, and Key Determinants

Cancer continues to pose one of the greatest global health challenges, accounting for an estimated 20 million new cases and 9.7 million deaths in 2022. These numbers highlight the immense scale of the disease, with current projections indicating that one in five individuals will develop cancer during their lifetime, and one in nine men and one in twelve women will ultimately die from it. Such statistics not only reflect the widespread prevalence of cancer but also emphasize its profound impact on survival and quality of life worldwide. The distribution of cancer incidence and mortality is far from uniform. Considerable regional differences exist, shaped by socioeconomic status, lifestyle behaviors such as smoking and diet, occupational and environmental exposures, and access to healthcare. High-income countries often report higher overall incidence rates due to more comprehensive screening and longer life expectancy, whereas low- and middle-income countries face disproportionately higher mortality rates because of limited early detection programs and restricted access to effective treatments. According to Globocan [1,2], lung cancer remains the most frequently diagnosed malignancy worldwide, with 2.5 million new cases (12.4%) and continues to be the leading cause of cancer-related deaths, responsible for 1.8 million fatalities (18.7%). Among women, breast cancer stands out as the most common diagnosis (11.6%) and ranks as one of the major causes of death, underscoring the urgent need for targeted prevention and therapeutic strategies. Colorectal cancer ranks third in incidence (9.6%) and second in mortality (9.3%), reflecting its significant global health burden. Other leading cancers include prostate cancer (7.3%), stomach cancer (4.9%), and liver cancer (7.8%). Gender-specific patterns are also noteworthy. In men, lung cancer continues to dominate both incidence and mortality, whereas in women, breast cancer remains the leading concern. These distinctions highlight the importance of tailoring prevention, screening, and treatment strategies to address gender-related differences in cancer biology, risk factors, and healthcare access. Given these rising numbers, it is increasingly important to recognize not only the primary burden of cancer but also its systemic complications, such as cachexia and cancer-induced cardiac dysfunction, which substantially worsen outcomes and demand integrated approaches to care.

### 1.2. Regional Disparities and the Role of Lifestyle and Environmental Factors

Cancer incidence and mortality differ markedly across regions, reflecting disparities in healthcare infrastructure, diagnostic capabilities, socioeconomic development, and population demographics. High-income regions, such as Australia and New Zealand, consistently report the highest incidence rates reaching 507.9 per 100,000 men and 410.5 per 100,000 women. These elevated figures are influenced by several factors, including longer life expectancy, greater availability of advanced diagnostic technologies, and lifestyle-related risks such as obesity, sedentary behavior, and alcohol consumption. Improved screening programs also contribute to higher detection rates, which, while beneficial for early intervention, inflate reported incidence figures compared to less developed regions. By contrast, regions such as Western Africa and South-Central Asia report significantly lower cancer incidence rates. However, these numbers often underestimate the true burden due to limited healthcare access, lack of diagnosis, and incomplete cancer registries. Younger population structures in these regions also contribute to the comparatively lower incidence, but mortality rates can be disproportionately high because cancers are frequently detected at advanced stages, when treatment options are limited or less effective.

Regional variations in cancer risk are strongly tied to exposure to modifiable factors. Tobacco use remains the leading preventable cause of cancer worldwide, with smoking and smokeless tobacco contributing heavily to lung, oral, and other cancers. Rising obesity rates, coupled with poor dietary habits and physical inactivity, further accelerate the incidence of multiple malignancies, including colorectal, breast, and endometrial cancers. Chronic infections also play a major role in shaping regional cancer burdens; for example, hepatitis B and C drive liver cancer rates in Asia and Africa, while human papillomavirus (HPV) is a dominant cause of cervical cancer in low and middle-income countries. Environmental exposures, such as air pollution, industrial chemicals, and asbestos, add another layer of risk, particularly in urban and industrialized regions. Finally, inherited genetic predispositions, while less modifiable, interact with these environmental and lifestyle factors to further shape cancer incidence across populations. Looking ahead, the global cancer burden is projected to nearly double, reaching an estimated 35 million new cases annually by 2050. This anticipated surge underscores an urgent need for stronger cancer control strategies worldwide. Expanding equitable access to healthcare, strengthening diagnostic and treatment capabilities, implementing lifestyle-based preventive measures, and addressing environmental and infectious contributors are essential steps toward mitigating the rising tide of cancer [2]. As mentioned above, the global cancer burden is projected to nearly double annually by 2050. This anticipated surge underscores an urgent need for stronger cancer control strategies worldwide. Expanding equitable access to healthcare, strengthening diagnostic and treatment capabilities, implementing lifestyle-based preventive measures, and addressing environmental and infectious contributors are essential steps toward mitigating the rising tide of cancer [2]. Importantly, these regional and lifestyle-driven disparities not only influence cancer incidence and survival but also shape the prevalence and severity of secondary complications such as cachexia and cancer-induced cardiac dysfunction, reinforcing the need for a holistic approach that integrates cancer treatment with management of comorbid conditions.

## 2. Cancer and Organ Dysfunction: Pathophysiology and Systemic Implications

Cancer affects not only the primary origin site but also has widespread impacts on nearly every tissue and organ in the body. This organ dysfunction results from a combination of direct tumor damage, systemic metabolic disturbances, and toxic effects of cancer treatments, together worsening both morbidity and mortality. Cachexia, a key feature of cancer-related metabolic decline, leads to severe loss of muscle and fat tissue, contributing to dysfunction in multiple organs, especially the heart and liver [3]. Driven by inflammatory cytokines and metabolic imbalances, cachexia exemplifies how cancer imposes a systemic burden on the body. Sarcopenia, which is particularly common in older cancer patients, adds to this burden by decreasing muscle mass and strength. This decline not only reduces physical ability but also disrupts metabolism, leading to insulin resistance and altered drug metabolism, which can complicate treatment [4]. Endocrine imbalances and the development of metabolic syndrome, often secondary to cancer, further contribute to organ dysfunction. Brown and Scherer [5] highlighted how changes in adipose tissue in cancer patients promote features of metabolic syndrome, including insulin resistance and dyslipidemia, which increase the risk of cardiovascular disease. Additionally, systemic amyloidosis can cause abnormal amyloid deposits in the heart, kidneys, and liver, thereby leading to progressive organ failure [6]. Neurological complications are also common in cancer. The disease’s interaction with the nervous system can lead to neuropathic pain, cognitive decline, and enhanced tumor growth, all of which negatively affect a patient’s quality of life [7]. Beyond the disease itself, cancer treatments also significantly impact organ health. Chemotherapy and radiation often cause gastrointestinal side effects such as mucositis, which interferes with nutrient absorption and increases infection risk [8]. Klassen et al. [9] described how musculoskeletal side effects of cancer therapies, particularly in breast cancer patients, impair mobility and hinder daily activities. Moreover, cancer-associated fibroblasts (CAFs) within the tumor microenvironment promote tumor growth and subsequent metastasis, hence further accelerating the decline of organ function throughout the body [10]. There is quite overlap between cancer-induced and therapy-induced toxic effects on various organs. However, some difference lies between cancer-induced and therapy-induced toxic effects (Table 1).

### 2.1. Impact of Cancer on Cardiac Function

Cancer-induced cardiac dysfunction is well established and results from both the physiological stress imposed by the malignancy itself and the cardiotoxic effects of cancer treatments. Cancer therapy-related cardiac dysfunction (CTRCD), often defined by a measurable reduction in left ventricular ejection fraction (LVEF), affects approximately 10% of patients receiving chemotherapy or radiation. The use of anthracyclines and trastuzumab significantly elevates this risk, with patient-specific factors such as advanced age, existing cardiovascular disease, and female sex modulating the susceptibility [11]. Treatment-related risk factors also play a critical role and include the cumulative dose of anthracyclines and the simultaneous administration of other cardiotoxic agents, and exposure to mediastinal radiation [12]. In addition to direct cardiotoxicity, systemic inflammation and oxidative stress associated with cancer further impair the cardiac function. Tumor-derived cytokines exert significant stress on myocardial tissue, while elevated levels of reactive oxygen species (ROS) cause oxidative damage to cardiac cells, contributing to a progressive decline in cardiac performance [13].

### 2.2. Pathophysiology of Heart Failure

Cancer-induced cardiac failure represents a complex and multifaceted syndrome that arises from the convergence of several mechanisms, including direct cardiotoxic effects of cancer therapies, immune-mediated injury, infiltrative diseases, systemic metabolic disturbances, and paraneoplastic phenomena. Among the most studied culprits are anthracyclines and trastuzumab agents that serve as prototypes for distinct types of cardiomyopathies. Anthracyclines cause a dose-dependent and often progressive injury through mechanisms linked to oxidative stress and mitochondrial dysfunction, whereas trastuzumab induces a largely reversible cardiomyopathy by disrupting HER2 signaling pathways [14].

A newer and particularly concerning entity is immune checkpoint inhibitor-associated myocarditis, which carries a high mortality rate and demands rapid recognition and aggressive immunosuppressive therapy [15]. In parallel, infiltrative disorders such as cardiac amyloidosis especially transthyretin (ATTR) and light chain (AL) subtypes are increasingly diagnosed in cancer patients, particularly those with monoclonal gammopathies or advanced age [6]. The advent of advanced non-invasive imaging modalities and targeted agents like tafamidis has revolutionized amyloidosis management, emphasizing the importance of early detection and disease-specific intervention. Beyond direct cardiotoxicity, systemic factors play a critical role. Conditions such as obesity and metabolic syndrome contribute to a chronic pro-inflammatory and pro-fibrotic environment, which not only predisposes patients to cardiac dysfunction but can also amplify the cardiotoxic effects of cancer therapies [16]. Recognizing this interconnected risk landscape, the Heart Failure Society of America has stressed the need for integrated cardio-oncology care, advocating for systematic risk stratification, longitudinal surveillance, and individualized treatment plans [17]. Recent data further suggest that SGLT2 inhibitors, already established in heart failure (HF) with reduced ejection fraction, may hold promise for improving outcomes in therapy-related cardiac dysfunction, though confirmatory trials are still required [18].

**Figure 1:**
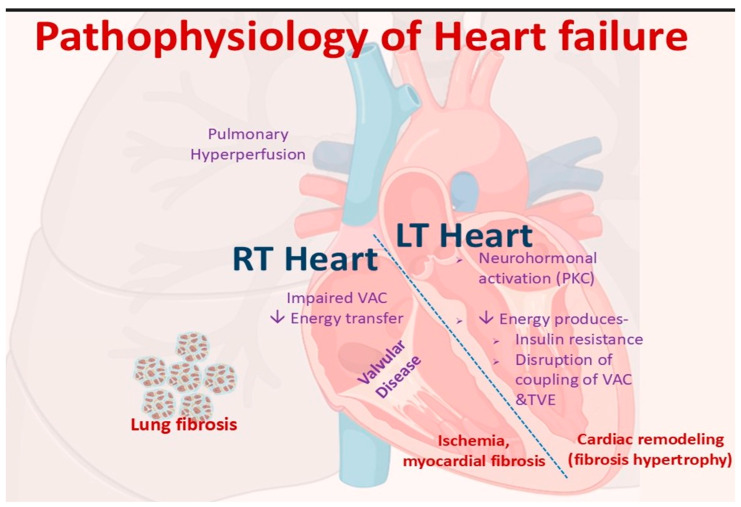
Schematic representation of pathophysiology showing the mechanisms underlying left and right heart failure: Neurohormonal activation, decreased energy production, insulin resistance, and disruption of ventriculo-arterial coupling are characteristic features of left heart failure (LT Heart), which leads to myocardial fibrosis, ischemia, and cardiac remodeling (fibrosis, hypertrophy). With compromised ventriculo-arterial coupling and energy transmission. Right heart failure (RT Heart) frequently occurs after pulmonary hyperperfusion or subsequent lung disease (such as lung fibrosis). RT heart: right heart, LT heart: left heart, VAC: ventriculo-arterial connection, TVE: tricuspid valve endocarditis, and PKC: protein kinase C.

Despite advances in understanding chemotherapy and amyloidosis-associated cardiac failure, knowledge gaps persist. In particular, the mechanisms underlying immune-related and paraneoplastic cardiomyopathies remain poorly defined, and the heterogeneity of cancer patient populations continues to limit the generalizability of available evidence. Addressing these gaps requires focused mechanistic studies and rigorously designed randomized clinical trials to establish effective preventive and therapeutic strategies. Collectively, these insights highlight the need for a comprehensive, mechanism-driven approach to managing cancer-induced HF (Figure 1). Future research should continue to unravel the diverse pathways of myocardial dysfunction while pursuing novel, targeted interventions capable of addressing the root causes of heart failure. Importantly, understanding these mechanisms is also critical for appreciating how cardiac dysfunction interconnects with broader cancer complications such as cachexia, where overlapping metabolic and inflammatory pathways further compromise patient survival and quality of life (QoL).

## 3. Mechanisms Underlying the Pathophysiology of Cancer-Induced Cardiac Dysfunction

### 3.1. Role of Cytokines

Cytokines play a central role in mediating cancer-induced cardiac dysfunction with emerging evidence linking their dysregulation to cardiotoxicity, metabolic disturbances, and structural heart damage in cancer patients. The pro-inflammatory cytokine milieu, often amplified by chemotherapy and tumor-derived signals, drives myocardial inflammation, fibrosis and impaired contractile function. Quagliariello et al. [19] demonstrated that empagliflozin, a sodium glucose co-transporter 2 (SGLT-2) inhibitor significantly alleviated doxorubicin-induced cardiotoxicity in non-diabetic mice. This cardio-protection was associated with a marked reduction in pro-inflammatory cytokines such as IL-1β, IL-6 and TNF-α alongside improvements in myocardial strain and decreased cardiac fibrosis. These findings suggest that modulation of cytokine expression may be an effective strategy to mitigate chemotherapy induced cardiac injury. Building on this, Zhou et al. [20] identified IL-1α as a key pathogenic mediator in daunorubicin-induced cardiotoxicity in patients with acute myeloid leukemia (AML). The study revealed that tumor-derived IL-1α disrupts cardiac energy metabolism and contributes to ventricular dysfunction. Importantly, treatment with an anti-IL-1α neutralizing antibody restored cardiac function in AML bearing mice receiving chemotherapy emphasizing IL-1α’s critical role in cancer-associated cardiac decline.

More broadly, Hanna [21] reviewed the detrimental effects of chronic cytokine elevation specifically TNF-α, IL-1, and IL-6 in the context of heart failure. These cytokines contribute to adverse cardiac remodeling, myocardial fibrosis and impaired contractility, and the effects that are often exacerbated in cancer patients undergoing cytotoxic therapies. The cardiotoxic influence of cytokines is not limited to structural damage but it also includes interference with signaling pathways essential for cellular survival and repair. Rolski [22] provided further insight into the dual role of TNF-α in cardiac inflammation and myocarditis. While TNF-α facilitates the recruitment of inflammatory cells and amplifies immune responses in the myocardium, it simultaneously induces apoptosis in activated T cells, limiting the extent of inflammation. This duality highlights the complexity of cytokine modulation in therapeutic contexts suggesting that while suppression may benefit heart function, overly aggressive inhibition could disrupt immune homeostasis. Increased circulating levels of TNF-α, IL-1, and IL-6 in cancer patients have also been implicated in muscle wasting including myocardial atrophy through activation of proteolytic pathways and inhibition of anabolic signaling via the Akt/mTOR pathway. These changes contribute to reduced cardiac mass and function. Belloum et al. [23] showed that elevated TNF-α and IL-6 levels correlate with the severity of cardiac dysfunction, reinforcing the central role of inflammatory cytokines in cancer-associated cardiomyopathy. Beyond their cardiac effects, these cytokines also contribute to oncogenesis. Heart failure is associated with the overproduction of pro-inflammatory cytokines, fostering a systemic inflammatory environment conducive to tumor initiation and progression. Cuomo [24] reports that TNF-α activates the NF-κB signaling pathway which promotes cancer cell survival and proliferation while concurrently aggravating cardiac dysfunction through enhanced cardiomyocyte apoptosis and fibrosis. Supporting this dual therapeutic potential, Quagliariello et al. [19] found that empagliflozin not only improved cardiac function but also significantly suppressed pro-inflammatory cytokine expression in doxorubicin-treated mice. These results position SGLT-2 inhibitors as a promising class of agents capable of attenuating cytokine-mediated cardiac injury in cancer patients, offering cardio-protection alongside potential systemic anti-inflammatory benefits.

### 3.2. Role of Reactive Oxygen Species

Reactive oxygen species (ROS), including free radicals such as superoxide (O_2_•^−^) and non-radical molecules like hydrogen peroxide (H_2_O_2_) are natural byproducts of cellular metabolism. Under physiological conditions, ROS act as important signaling molecules regulating diverse cellular processes such as proliferation, apoptosis and immune responses [25,26,27]. However, when produced in excess or inadequately neutralized by endogenous antioxidant systems then ROS contributes to oxidative stress, a major pathological driver in numerous diseases including cancer-induced cardiac dysfunction [27,28]. Among cancer therapies, the cardiotoxic potential of ROS is particularly pronounced with anthracyclines such as doxorubicin (DOX). DOX-induced cardiotoxicity has been largely attributed to ROS production through redox cycling and disruption of mitochondrial iron homeostasis, leading to mitochondrial dysfunction and cardiomyocyte injury [29,30]. Kong et al. [30] reinforced this mechanism showing that DOX promotes acute cardiac injury by activating oxidative stress and programmed cell death pathways including both apoptosis and ferroptosis [31]. Fang et al. [32] introduced ferroptosis, a novel iron-dependent form of cell death as a key ROS-mediated mechanism underlying DOX-induced cardiomyopathy. Their study demonstrated that pharmacological inhibition of ferroptosis with ferrostatin-1 significantly attenuates cardiac injury, highlighting lipid peroxidation as a pivotal step in ROS-driven cardiac damage. Complementing these findings, Li et al. [33] explored the role of acetylation in regulating oxidative stress indicating that epigenetic modifications can influence ROS production and cardiomyocyte susceptibility to injury.

**Figure 2:**
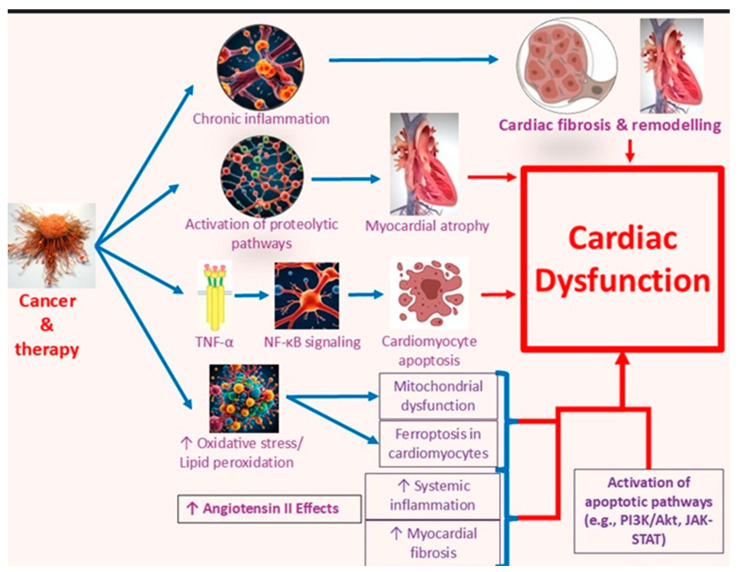
Crosstalk between cytokines, reactive oxygen species (ROS), and angiotensin II (Ang II) in the development of cardiac dysfunction in cancer patients: Ang II induces immune cell activation, which results in the release of cytokines and the generation of ROS, which causes oxidative stress, inflammation, and mitochondrial damage. Chemotherapy further exacerbates these effects, creating a vicious cycle that leads to cardiac remodeling, fibrosis, and dysfunction. Antioxidants, cytokine inhibitors, and angiotensin receptor blockers are examples of targeted therapy that may break this loop and lower the risk of cardiotoxicity. TNF-α: tumor necrosis factor alpha, NF-kB: nuclear factor kappa-B, PI3K: Phosphoinositide 3-kinase, Akt: protein kinase B, JAK: Janus kinase, STAT: signal transducer and activator of transcription.

Several strategies have been proposed to mitigate ROS-induced cardiotoxicity. Sheibani et al. [34] provided a comprehensive review of preclinical interventions including direct antioxidants and agents that inhibit ROS-generating enzymes, supporting their potential as cardioprotective therapies. Al-Hussaniy et al. [28] further reported on the antioxidant properties of resveratrol and various herbal compounds suggesting alternative approaches to limit ROS-related cardiac injury. The detrimental effects of ROS are not confined to direct chemotherapy induced damage as elaborated by Bindu et al. [35] highlighting that the oxidative stress triggered by non-steroidal anti-inflammatory drugs (NSAIDs) thus emphasizing that pharmacologic agents beyond chemotherapeutics can also compromise cardiac function through ROS-mediated pathways. In parallel, Wang et al. [36] identified tumor-derived extracellular vesicles as contributors to systemic oxidative stress particularly via hepatic metabolic disruption, which may amplify the cardiotoxic effects of chemotherapy. Among emerging interventions, melatonin has shown considerable promise due to its dual ability to directly scavenge ROS and enhance endogenous antioxidant defenses. Reiter et al. [37] demonstrated melatonin’s efficacy in reducing oxidative damage and improving cardiac resilience, hence making it a compelling candidate for ROS-targeted cardio-protection. Moreover, the shared risk factors and overlapping pathophysiological pathways of cardiovascular disease and cancer as discussed by Koene et al. [38], further underscore the importance of oxidative stress management in preventing cancer-related cardiotoxicity. Specifically, ROS-mediated inflammation and mitochondrial dysfunction act as common denominators linking the two disease entities. In short, the evidence firmly establishes ROS as the central mediator of oxidative damage, mitochondrial impairment and cell death in cardiomyocytes that are exposed to chemotherapeutic agents. While antioxidant therapies and ROS modulating agents have shown encouraging results in preclinical models, their clinical translation remains limited [39] (Figure 2). Ongoing research is needed to identify safe, effective and translatable interventions that can mitigate ROS driven cardiotoxicity in cancer patients.

The interplay between reactive oxygen species (ROS) and cellular signaling pathways plays a pivotal role in the development of cancer-induced cardiac dysfunction. For example, anthracycline-based chemotherapies, widely used in cancer treatment, are well known for their cardiotoxic side effects that are primarily mediated by ROS generation [30]. These oxidative stressors activate multiple intracellular signaling cascades that regulate cardiomyocyte survival, apoptosis, inflammation and metabolic function. One of the key pathways affected by ROS is the PI3K/Akt signaling axis which governs cellular survival and apoptosis in cardiomyocytes. Prem Et Al. [39] demonstrated that the flavonoid fisetin attenuates myocardial Ischemia–Reperfusion injury by modulating this pathway thereby highlighting its protective role against ROS-induced cardiac damage. Similarly, the JAK-STAT pathway is another critical target of ROS-mediated modulation. Fisetin’s ability to attenuate JAK-STAT signaling as reported by Prem Et Al. [39] suggests a broader anti-inflammatory and anti-apoptotic effect in oxidative environments. Furthermore, Holmström and Finkel [40] confirmed the redox sensitivity of these signaling pathways showing that ROS can reversibly oxidize cysteine residues on signaling proteins, thereby modulating the activity of cascades such as JAK-STAT and MAPK. These redox-dependent modifications underscore the dual role of ROS, functioning both as messengers and as mediators of damage, depending on their concentration and cellular context.

Another essential regulator of the oxidative stress response is nuclear factor erythroid 2–related factor 2 (Nrf2), a transcription factor that orchestrates the cellular antioxidant defense system. Chen and Maltagliati [41] emphasized Nrf2′s role in upregulating genes involved in detoxification and antioxidative protection. While Nrf2 activation can confer resilience against ROS mediated injury, its effectiveness is highly dependent on the redox environment. Excessive ROS may overwhelm this protective mechanism leading to apoptosis and cardiac dysfunction. Sirtuins, particularly SIRT1 and SIRT6 have also emerged as vital regulators in this context. D’Onofrio et al. [42] showed that SIRT1 and SIRT6 mitigated oxidative stress by deacetylating transcriptional regulators and attenuating inflammatory signaling. Through this process, sirtuins enhance the expression of protective genes and inhibit pro-apoptotic factors thereby promoting cardiomyocyte survival and preserving cardiac function under ROS-induced stress. Ferroptosis, a regulated form of cell death characterized by iron-dependent lipid peroxidation is intricately linked to ROS activity. Xie et al. [43] identified ROS accumulation as a driving force behind ferroptotic cell death in cardiomyocytes and subsequent cardiac dysfunction. Hence, inhibiting ferroptosis via iron chelators or lipid peroxidation inhibitors can present a promising therapeutic avenue to prevent ROS-mediated cardiac damage in cancer patients undergoing chemotherapy. Additionally, inducible nitric oxide synthase (iNOS) is upregulated in response to inflammatory stimuli and contributes to ROS production. Cinelli et al. [44] discussed how dysregulated iNOS activity exacerbates oxidative stress and amplifies cardiac injury. As a result, targeting iNOS could serve as a strategy to reduce ROS levels and protect against cardiac dysfunction in cancer patients.

The interaction between metabolic pathways and ROS also significantly impacts cardiac health. Xie et al. [45] described how metabolic reprogramming in cardiomyocytes influences mitochondrial function and ROS production. Changes in metabolism can either exacerbate or alleviate oxidative stress, thus affecting the heart’s resilience to cancer-induced damage. However, the precise mechanisms through which metabolic shifts modulate ROS signaling in cardiac tissues remain to be fully elucidated. Together, these studies highlight the complex role of ROS in activating various signaling pathways including PI3K/Akt, JAK-STAT, Nrf2, SIRT1/SIRT6 and MAPK, all of which contribute to cardiac dysfunction during cancer treatment. Therefore, modulating these pathways presents a promising approach to developing cardioprotective strategies aimed at reducing ROS-induced damage in cancer patients. Nonetheless, the translational potential of these findings is limited by the delicate balance between the physiological and pathological roles of ROS. Therapeutic interventions must carefully regulate ROS levels to optimize their beneficial signaling effects while minimizing harmful outcomes. Additionally, individual differences in ROS production and antioxidant capacity call for personalized strategies to effectively address cancer-related cardiac dysfunction. We believe that future research should aim to clarify the molecular interactions within these signaling networks and identify biomarkers that predict susceptibility to ROS driven cardiac damage. These insights will pave the way for targeted therapies that protect cardiac function without compromising the anticancer effects of ROS inducing treatments.

### 3.3. Role of Angiotensin II

Angiotensin is a key molecule implicated in pulmonary hypertension and is upregulated in the circulation of cancer patients. The involvement of angiotensin II in cancer-related cardiac dysfunction is well documented across a wide spectrum of clinical and mechanistic studies. A systematic review and meta-analysis by Gao et al. [46] which included 15 randomized controlled trials (RCTs) encompassing 1977 breast cancer patients undergoing anthracycline and trastuzumab therapy revealed that angiotensin-converting enzyme inhibitors (ACEIs) and angiotensin receptor blockers (ARBs) significantly preserved left ventricular ejection fraction (LVEF). The standardized mean difference (SMD) was 0.556 (95% CI: 0.299–0.813), indicating a robust cardioprotective effect. Similarly, Lewinter et al. [47] evaluated nine RCTs involving 1362 patients and demonstrated that both beta-blockers and ACEIs/ARBs attenuated LVEF decline during anthracycline and trastuzumab treatment with mean differences of 2.4 and 1.5, respectively. However, findings from Goulas et al. [48] provided a contrasting view. Their meta-analysis of six RCTs focusing on the primary prevention of trastuzumab-associated cardiotoxicity found that although ACEIs/ARBs resulted in a modest but statistically significant improvement in LVEF, they did not significantly reduce the overall incidence of cardiotoxicity (OR = 0.92, 95% CI: 0.54–1.56). These results suggest that while angiotensin II blockade may preserve cardiac function, but its role in preventing the onset of cardiotoxicity remains limited.

Further supporting the protective role of RAAS inhibitors, Keshavarzian et al. [49] conducted a meta-analysis of 17 studies including 2674 patients and reported that ACEIs and beta-blockers significantly prevented LVEF decline over 6- and 12-month chemotherapy periods. This highlights the sustained benefits of RAAS inhibition in limiting angiotensin II-mediated cardiac injury. In childhood cancer survivors, Cheuk et al. [50] updated the Cochrane review on medical interventions for anthracycline induced cardiotoxicity. Enalapril, an ACEI, showed temporary improvements in cardiac function but was associated with side effects such as dizziness and hypotension, emphasizing the delicate risk-benefit balance in younger patients. In adult cohorts, Yun et al. [51] found that beta-blockers and angiotensin antagonists were associated with improved LVEF preservation post chemotherapy with a mean difference of 6.06% (95% CI: 0.54–11.58), further underscoring the efficacy of RAAS inhibition in adults receiving high dose anthracycline regimens. Mechanistically, McGowan [29] and Caspani et al. [52] elucidated how angiotensin II exacerbates cardiac dysfunction by promoting oxidative stress, inflammation and myocardial fibrosis. These pathophysiological processes strengthen the rationale for targeting angiotensin II as a preventive strategy.

Henriksen [53] added that angiotensin II contributes to topoisomerase 2β inhibition, thereby activating pro-apoptotic signaling pathways implicated in anthracycline induced cardiotoxicity. Expanding the clinical scope, Mennuni et al. [54] discussed the role of RAAS activation in hypertensive renal injury which often coexists with cardiac dysfunction in cancer patients, further complicating the cardiovascular landscape. Overall, these studies converge on the central role of angiotensin II in mediating cardiac dysfunction through multiple molecular mechanisms including myocardial fibrosis, hypertrophy, inflammation and apoptosis. The activation of the renin–angiotensin–aldosterone system (RAAS) elevates angiotensin II levels, which act on angiotensin II type 1 receptors (AT1R) in cardiac myocytes and fibroblasts initiating pro-fibrotic and pro-inflammatory signaling cascades. These cascades culminate in adverse cardiac remodeling, reducing cardiac output and ultimately heart failure. Pharmacological interventions targeting angiotensin II, particularly ACEIs and ARBs, have thus emerged as key strategies for mitigating cardiotoxicity in cancer patients treated with RAAS-activating agents like anthracyclines and trastuzumab. The consistent preservation of LVEF across multiple studies affirms their cardioprotective potential. However, the variability in outcomes, especially regarding the prevention of cardiotoxicity onset, does suggest the need for personalized treatment approaches based on patient-specific factors such as age, comorbidities, cancer type and chemotherapy regimen. Moreover, the side effects associated with RAAS inhibitors, including hypotension, electrolyte imbalances and renal complications, necessitate careful patient monitoring and dosage adjustments. Future research should focus on optimizing the timing and dosing strategies, exploring synergistic combinations with other cardioprotective agents and identifying predictive biomarkers to tailor angiotensin II-targeted therapies more precisely. Finally, in cancer cachexia, cardiac dysfunction is further driven by metabolic derangements including enhanced cardiac muscle proteolysis and mitochondrial dysfunction. This catabolic state is exacerbated by inflammatory cytokines such as IL-6, IL-1, TNFα and TGFβ. Elevated levels of angiotensin II in cachectic patients may contribute to cardiac impairment by reducing circulating IGF-1 and modulating MMP activation, as reported by Saha et al. [55]. This multifactorial interplay reinforces the need to consider angiotensin II not only as a mediator of cardiotoxicity but also as a potential therapeutic target in the broader metabolic context of cancer-induced cardiac dysfunction.

### 3.4. Role of Gut Microbiota and Its Metabolites

The intricate relationship between gut microbiota and cardiac health in cancer patients has garnered increasing attention, with emerging evidence highlighting how microbial composition and its metabolites influence systemic physiological processes. Hou et al. [56] emphasized that dysbiosis an imbalance in gut microbial communities can trigger chronic inflammation and metabolic disturbances, both of which are critical contributors to cardiovascular disease (CVD) pathogenesis. This is particularly relevant in cancer patients, where therapies such as chemotherapy and radiation further disrupt the gut microbial ecosystem, amplifying systemic inflammation and metabolic dysfunction. For example, probiotics have been explored for their cardioprotective potential. Taslim et al. [57] conducted a systematic review and meta-analysis of randomized controlled trials involving 366 participants to evaluate the role of probiotics in preventing heart failure (HF) and attenuating cardiac remodeling after myocardial infarction (MI). While no significant improvements were observed in left ventricular ejection fraction (LVEF) or high-sensitivity C-reactive protein (hs-CRP) levels, notable reductions were reported in total cholesterol (*p* = 0.01) and uric acid (*p* = 0.014). These findings suggest that although probiotics may not directly enhance cardiac function, they confer metabolic benefits that may indirectly support cardiovascular health. However, the limited sample sizes and number of studies underscore the need for more robust clinical trials to validate these effects. Further, dietary components, particularly fiber and bioactive compounds, play a pivotal role in modulating gut microbiota and cardiovascular outcomes. Anthocyanins, a class of flavonoids found in various fruits and vegetables, have been shown to promote microbial diversity and stimulate the growth of beneficial bacteria such as Bifidobacterium and Lactobacillus [58,59]. These bacteria produce short-chain fatty acids (SCFAs) and other metabolites that improve endothelial function and reduce oxidative stress, key factors in the development of CVDs. In cancer patients, who often experience therapy-induced microbial compromise, anthocyanin-rich diets may help restore microbial balance and mitigate cardiac risk.

**Figure 3:**
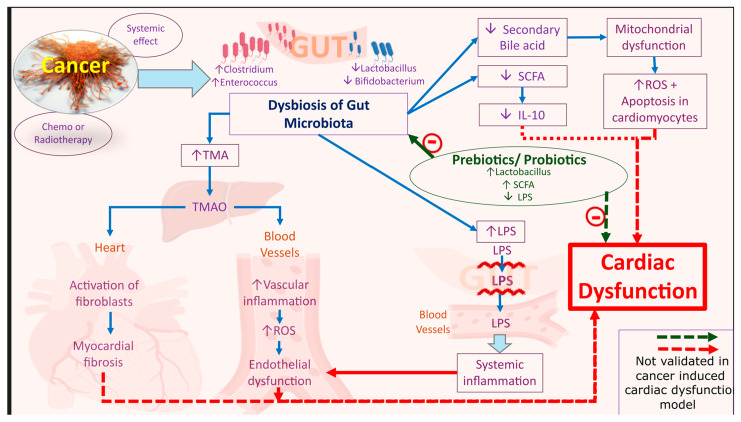
The pathophysiology of cardiac dysfunction in cancer patients is significantly influenced by gut microbiota and its metabolites. Gut dysbiosis brought on by cancer and its treatments can result in elevated inflammation, altered metabolism, and mitochondrial dysfunction, all of which can exacerbate heart damage. While short-chain fatty acids (SCFAs) have protective benefits via modulating inflammation and metabolism, microbial metabolites such as trimethylamine N-oxide (TMAO) encourage vascular inflammation and endothelial dysfunction. Probiotics could reduce the cardiotoxicity caused by chemotherapy, which gets worse with dysbiosis. In cancer patients, the gut-heart axis affects cardiac dysfunction through metabolic and inflammatory mechanisms. TMA: Thrombotic Microangiopathy, TMAO: trimethylamine N-oxide, ROS: reactive oxygen species, SCFA: short chain fatty acid, IL-10: interleukin-10, LPS: lipopolysaccharide. Mechanism showing dotted lines have not been validated in cancer induced cardiac dysfunction model.

The interplay between dietary interventions, gut microbiota, and cardiac health is further demonstrated by Qi et al. [60], who investigated microbial metabolites such as bile acids and interleukin-22 (IL-22) in regulating systemic inflammation and metabolism. In dysbiosis, altered bile acid metabolism can increase intestinal permeability and systemic endotoxin exposure, activating inflammatory cascades that adversely affect cardiac tissue. Interventions that modulate bile acid profiles or IL-22 production, whether through diet or pharmacologic agents, may help preserve cardiac function. Circadian rhythm, though less recognized, also significantly influences microbial regulation. Voigt et al. [61] showed that disruptions caused by irregular treatment schedules or psychological stress can alter gut microbiota, exacerbate inflammation, and impair metabolic regulation, ultimately increasing cardiovascular risk. Restoring circadian alignment through consistent meal timing and sleep patterns may stabilize gut microbial communities and reduce inflammation, supporting cardiac health. Therapeutic strategies such as probiotics, prebiotics, synbiotics, and targeted dietary modifications have shown promise in restoring microbial balance and enhancing metabolic homeostasis [62,63,64]. Bulut et al. [65] demonstrated in a hyperglycemic rat model of myocardial Ischemia–Reperfusion injury that synbiotic supplementation improved gut microbiota composition, reduced systemic inflammatory cytokines (TNF-α, IL-6), lowered cardiac injury biomarkers (CK-MB, cTnI, *p* < 0.05), and alleviated anxiety-like behavior, suggesting relevance to the psycho-neuroimmune axis in cancer patients. While this study supports the potential of gut microbiota modulation in reducing cardiac injury under metabolic stress, it does not directly model cancer-induced cardiac dysfunction. In short, the current literature points to a compelling link between gut microbiota and cardiac health in cancer patients. Interventions aimed at reestablishing microbial harmony including probiotics, dietary fiber, anthocyanins, and synbiotics do offer promising avenues for cardiac protection (Figure 3). Nevertheless, the field remains limited by a lack of large-scale, cancer-specific clinical trials. Future research should prioritize randomized controlled studies in oncologic populations to clarify mechanistic pathways and establish evidence-based guidelines for gut microbiota modulation in improving cardiovascular outcomes. We believe that current understanding and targeting gut microbial dysbiosis may also have implications for mitigating downstream systemic complications, including cancer-induced cachexia and progressive cardiac dysfunction.

### 3.5. Role of Exosomes and Micrornas

Intercellular communication has emerged as a pivotal factor in both cancer progression and cardiovascular health. In this context, increasing attention has been directed toward the role of cancer cell-derived exosomes in modulating cardiac function, particularly in the pathophysiology of myocardial infarction (MI) and heart failure (HF), as outlined below, briefly.

#### 3.5.1. Exosomal Cargo and Its Cardiovascular Impact

Exosomes are small extracellular vesicles that carry a diverse array of bioactive molecules, including microRNAs (miRNAs), proteins, and lipids, which can significantly alter the behavior of recipient cells. In the context of cancer, tumor-derived exosomes have been implicated in promoting oxidative stress and inflammation in cardiac tissues, contributing to endothelial dysfunction and the exacerbation of conditions such as HF. Zhang et al. [66] provided a comprehensive review highlighting the diverse roles of exosomes in cardiovascular diseases (CVDs), including coronary artery disease, MI, and cardiomyopathies. These vesicles were shown to mediate critical pathological processes such as endothelial injury, apoptosis, oxidative stress, and inflammation. Notably, the non-coding RNA content of exosomes including miRNAs, long non-coding RNAs (lncRNAs), and circular RNAs (circRNAs) holds potential as diagnostic biomarkers due to their disease-specific expression profiles [67,68,69]. Additionally, the capacity of exosomes to serve as therapeutic delivery platforms opens new avenues for targeted cardiovascular treatments [70].

#### 3.5.2. MicroRNAs in Exosomal Cardio-Protection and Cardiotoxicity

MicroRNAs encapsulated in extracellular vehicles (EVs) have demonstrated promise both as biomarkers and as mediators of cardiotoxicity and cardio-protection. Beaumier et al. [69] investigated circulating EV-miRNAs as early indicators of doxorubicin-induced cardiotoxicity in a canine model. Although limited by a small sample size (*n* = 9), the study demonstrated notable changes in miRNA expression profiles following chemotherapy, underscoring the potential of EV-miRNAs for early detection of cardiac injury. Building on these important insights, Lee et al. [71] demonstrated that small extracellular vesicles derived from mesenchymal stem cells (MSC-sEVs) could help protect against doxorubicin-induced cardiomyopathy. The study specifically implicated miR-199a-3p in regulating the Akt-Sp1/p53 signaling axis, showing both in vitro and in vivo that MSC-sEVs convey cardio-protection via miRNA-mediated mechanisms. Related investigations by Xia et al. [72] and Li et al. [73] further highlighted the roles of miR-199a-3p and miR-133b, respectively, in mitigating cardiotoxicity, though these studies did not specifically focus on exosomal delivery from tumor cells.

## 4. Gaps in Knowledge and Future Directions

**Figure 4:**
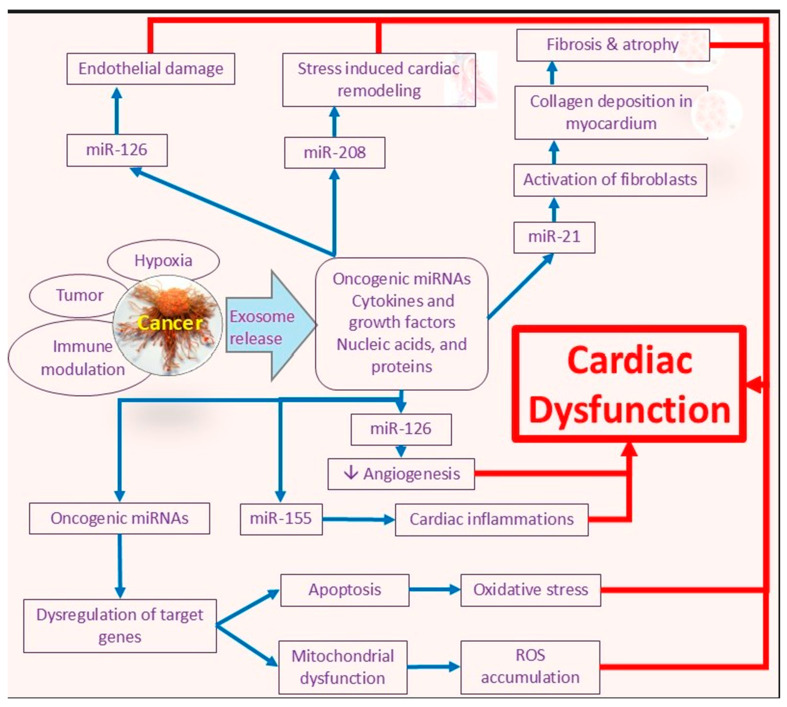
Mechanisms by Tumor-derived exosomes and microRNAs (miRNAs) contribute to cancer-induced cardiac dysfunction by intercellular communication with cardiac cells. Exosomes carry miRNAs, which can cause cardiomyocyte apoptosis, inflammation, fibrosis, and oxidative stress. miRNAs such as miR-126, miR-208, miR-21, and miR-155 modulate signaling pathways implicated in angiogenesis, stress-induced cardiac remodeling, cardiac hypertrophy, and fibrosis. Dysregulation of miRNAs exacerbates chemotherapy-induced cardiotoxicity, and exosomal miRNAs are emerging as biomarkers and possible therapeutic targets for preventing heart harm in cancer patients. miRNA: micro RNA, ROS: reactive oxygen species.

Despite these advancements, critical gaps remain in our understanding of how tumor-derived exosomal miRNAs directly affect cardiac myocytes. Elucidating these interactions could uncover novel therapeutic targets capable of minimizing cardiotoxicity without compromising the efficacy of cancer treatments. Furthermore, identifying the molecular signatures of tumor-derived exosomes may facilitate the development of predictive biomarkers for cardiac complications, thus advancing personalized approaches in oncology. Future research should focus on isolating and characterizing exosomes specifically derived from tumor cells, particularly in the context of chemotherapeutic exposure. Further, integration of high-throughput sequencing with functional cardiomyocyte assays may clarify causal mechanisms and uncover key signaling pathways. Validation of preclinical findings through clinical studies involving patient-derived samples is essential to ensure translational relevance. Moreover, employing multi-omics approaches including transcriptomics, proteomics, and metabolomics can enrich our understanding of the “tumor–heart axis” during cancer therapy (Figure 4). While the diagnostic and therapeutic potential of exosomes in cardiovascular contexts is promising, the regulatory mechanisms that govern their function remain poorly defined. Therefore, continued investigation is essential to delineate context-specific roles of exosomes across various pathological states. A more comprehensive understanding may ultimately drive the development of innovative diagnostic tools and targeted interventions to effectively manage cardiovascular complications in cancer patients.

## 5. Diagnosis of Cardiac Dysfunctions

The primary goal of cardio-oncology is to ensure that cancer patients can safely receive effective therapies while minimizing cancer treatment-related cardiovascular toxicity (CTR-CVT) throughout their care journey. Before initiating cancer therapies known to impact the heart, the cardio-oncology team should comprehensively assess and manage any preexisting cardiac conditions or risk factors. CTR-CVT encompasses a wide spectrum of complications, including HF, myocarditis, vascular toxicity (such as coronary artery disease, peripheral artery disease, stroke, transient ischemic attack, myocardial infarction, and acute coronary syndrome), arterial hypertension, and cardiac arrhythmias (e.g., QT prolongation, bradycardia, supraventricular tachycardia, ventricular arrhythmia, and atrial fibrillation). The optimal time to begin cardiovascular risk prevention in cancer patients is at the point of cancer diagnosis, before initiating treatment. Early evaluation allows the healthcare team to consider cardiac risks when designing individualized cancer therapy plans, educating patients on potential cardiovascular complications, personalize monitoring schedules, and refer high-risk individuals to cardio-oncology specialists. These proactive measures help reduce cardiac complications, improve adherence to cancer treatment, and enhance overall survival [74]. Importantly, cardiovascular prevention strategies must be tailored to each patient, balancing the complexity of heart risk assessment with the need to avoid delaying essential cancer therapy. The selection of diagnostic tests including electrocardiography (ECG), cardiac biomarkers, and imaging modalities should be guided by the patient’s baseline cardiovascular risk and the specific cancer treatment regimen.

Cardiovascular Risk Assessment: A structured risk assessment is critical for estimating the likelihood of cardiotoxicity and guiding preventive interventions. Validated risk stratification tools that incorporate multiple clinical variables are recommended. While many existing scores are limited to specific cancer types, the HFA-ICOS risk assessment tool is particularly useful in oncology settings due to its simplicity and ease of use. Additionally, general cardiovascular risk calculators, such as SMART (Second Manifestations of Arterial Disease), ADVANCE (Action in Diabetes and Vascular Disease: Preterax and Diamicron-MR Controlled Evaluation), SCORE2 (Systematic Coronary Risk Estimation 2), ASCVD (Atherosclerotic Cardiovascular Disease), and U-Prevent, may help inform risk assessment, with the important caveat that cancer itself represents an independent risk factor for cardiovascular disease. The results of these assessments should be carefully communicated to patients and documented in clinical records to support future validation studies and facilitate personalized care [75,76].

Risk assessment through History taking and clinical examination: A comprehensive clinical history and physical examination are fundamental to baseline cardiovascular (CV) risk assessment in oncology patients. Patients should be stratified into two principal groups based on the presence or absence of pre-existing cardiovascular disease (CVD), which subsequently guides the selection of primary or secondary prevention strategies. Primary prevention applies to patients without prior CVD or cancer therapy-related cardiovascular toxicity (CTR-CVT), whereas secondary prevention is directed toward those with established or ongoing CVD or previous CTR-CVT. Evaluation of traditional, modifiable cardiovascular risk factors (CVRFs) is essential. When such risk factors are present, their current control and the efficacy of any ongoing treatment should be assessed to ensure optimal management during cancer therapy. Patients with existing CVD, diabetes, chronic kidney disease, or markedly elevated single risk factors should be immediately classified as high or very high risk. Family history of premature CVD, as well as lifestyle factors including smoking, alcohol consumption, physical inactivity, environmental pollution exposure, and frailty must also be considered due to their overlapping impact on both cancer progression and cardiovascular risk. Further, detailed documentation of prior cancers, exposure to cardiotoxic therapies, and cumulative drug doses is imperative. Additionally, patients should be screened for cardiac symptoms such as chest pain, exertional dyspnea, orthopnea, palpitations, and peripheral edema, which provide critical insight for further diagnostic evaluation. In the secondary prevention cohort patients with prior CVD, a more extensive assessment of disease severity, prior interventions, and current management is warranted. The risk–benefit discussions should be conducted in a multidisciplinary manner, ideally involving oncologists, cardiologists, and a dedicated cardio-oncology service. Comprehensive baseline CTR-CVT risk assessment should therefore integrate clinical history, physical examination findings, treatment-related exposures, electrocardiography (ECG), cardiac biomarkers, and imaging studies to generate an accurate and personalized risk profile, enabling safe and effective cancer therapy planning [77,78].

Electrocardiogram-based assessment: A baseline 12-lead electrocardiogram (ECG) is a widely accessible and essential tool for detecting pre-existing cardiovascular disease (CVD) in cancer patients. ECG can reveal a variety of abnormalities, including chamber enlargement, conduction defects, arrhythmias, ischemic changes, evidence of prior myocardial infarction, and low-voltage patterns. Interpretation should always be performed in the context of the patient’s overall clinical profile. Baseline ECG is particularly critical before initiating cancer therapies known to prolong the QT interval. In these cases, measurement of the corrected QT interval using Fridericia’s formula (QTcF) is recommended. If QTcF prolongation is detected at baseline, reversible causes such as electrolyte disturbances or interacting medications should be addressed, and evaluation for congenital long QT syndromes may be warranted. Specific ECG findings may also provide predictive value for arrhythmic complications during cancer therapy. For example, left atrial enlargement has been associated with an increased risk of atrial fibrillation (AF) in patients receiving ibrutinib. Similarly, atrioventricular (AV) conduction delays and the presence of premature atrial complexes before autologous hematopoietic stem cell transplantation (HSCT) have been linked to a higher incidence of atrial arrhythmias during treatment. Briefly, baseline ECG offers a simple yet informative assessment of cardiac status, serving as a cornerstone in cardio-oncology risk stratification and guiding monitoring strategies throughout cancer therapy [79,80].

Cardiac biomarkers-based assessment: The current evidence supporting the use of cardiac biomarkers for pre-treatment risk stratification of cancer therapy-related cardiovascular toxicity (CTR-CVT) remains limited, with most recommendations based on expert consensus. Four recent position statements from collaborative efforts among the Cardio-Oncology Study Group of the Heart Failure Association (HFA) of the European Society of Cardiology (ESC), the ESC Council of Cardio-Oncology (ESC-CCO), and the International Cardio-Oncology Society (ICOS) advocate for the use of cardiac troponins (cTnI or cTnT) and natriuretic peptides (BNP or NT-proBNP) for baseline cardiovascular risk assessment. These biomarkers may be particularly valuable for patients scheduled to receive therapies with high cardiotoxic potential, including anthracyclines, HER2-targeted agents, VEGF inhibitors (VEGFi), proteasome inhibitors (PI), immune checkpoint inhibitors (ICI), chimeric antigen receptor T-cell (CAR-T) therapy, and tumor-infiltrating lymphocyte (TIL) therapies. Baseline measurements can help identify patients at increased risk of cardiac injury who may benefit from early cardioprotective strategies. Baseline biomarker assessment is also crucial if serial monitoring is planned to detect subclinical myocardial injury during cancer treatment. Several studies have demonstrated the predictive value of cardiac biomarkers, particularly in patients receiving anthracyclines or trastuzumab. Elevated baseline cardiac troponin, especially high-sensitivity troponin I (hs-cTnI) and T (hs-cTnT), has been associated with a higher risk of developing left ventricular dysfunction (LVD). For instance, in women receiving trastuzumab, baseline hs-cTnI (>40 ng/L) or hs-cTnT (>14 ng/L) correlated with a fourfold increased risk of LVD and reduced likelihood of recovery, even with standard HF therapy. However, prior anthracycline exposure in many patients complicates interpretation, as elevated troponin may reflect cumulative pre-treatment myocardial injury rather than true baseline values [81]. Natriuretic peptides (NPs), including BNP and NT-proBNP, also serve as valuable tools for cardiovascular risk stratification. In multiple myeloma patients treated with carfilzomib, elevated baseline BNP (>100 pg/mL) or NT-proBNP (>125 pg/mL) strongly predicted cardiovascular adverse events (odds ratio; OR 10.8). Elevated NP levels have also been associated with all-cause mortality across several cancer types, suggesting subclinical cardiac injury related to disease burden. Nonetheless, some registries, such as CARDIOTOX, reported that baseline NT-proBNP and troponin levels did not consistently predict severe CTRCD, highlighting variability in prognostic utility. Emerging biomarkers including myeloperoxidase, CRP, galectin-3, growth differentiation factor-15 (GDF-15), microRNAs, placental growth factor, and others are under investigation for CTR-CVT risk stratification. While these markers show potential, current evidence does not support their routine clinical use, hence further research is required to validate these novel biomarkers and clarify their role in personalized cardiovascular risk assessment for cancer patients [82].

Cardiovascular imaging-based evaluation: Cardiovascular imaging plays a pivotal role in the baseline assessment of cancer patients by identifying subclinical cardiovascular disease (CVD), quantifying pre-existing cardiac abnormalities, and providing reference points for monitoring treatment-related cardiac changes and long-term follow-up. Transthoracic echocardiography (TTE) is the preferred initial imaging modality due to its ability to evaluate left ventricular (LV) and right ventricular (RV) function, chamber size, LV hypertrophy, regional wall motion abnormalities, diastolic function, valvular heart disease (VHD), pulmonary artery pressure (PAP), and pericardial disease. Baseline assessments should include left ventricular ejection fraction (LVEF) and global longitudinal strain (GLS), particularly in patients at moderate or high risk for CTR-CVT [83]. Three-dimensional (3D) echocardiography is recommended for accurate measurement of LVEF and cardiac volumes, while the two-dimensional (2D) Simpson’s biplane method is acceptable if 3D imaging is not feasible. Contrast agents should be used when image quality is suboptimal [84]. Cardiac magnetic resonance (CMR) serves as the preferred second-line modality when echocardiographic windows are inadequate [70]. Multigated acquisition (MUGA) scans remain a third-line option but should be minimized due to radiation exposure and limited functional information [85]. A borderline LVEF (50–54%) or reduced LVEF (<50%) at baseline is a recognized risk factor for CTR-CVT, particularly with anthracycline or trastuzumab therapy. Elevated indexed LV end-diastolic volume may also predict future major cardiac events. Because normal LVEF does not rule out early dysfunction, GLS measurement via speckle-tracking echocardiography is recommended, with a ≥15% relative reduction during treatment serving as a robust predictor of subsequent LVEF decline [86]. Global circumferential strain has potential as a predictive marker but is not yet recommended for routine clinical use due to limited evidence. Baseline LV diastolic dysfunction may be associated with future systolic impairment, though findings remain inconsistent [87].

For patients with pre-existing CVD (secondary prevention), a comprehensive TTE should assess baseline cardiac status and disease severity. In cases of poor echocardiographic quality or when detailed evaluation is required (e.g., hypertrophic cardiomyopathy), CMR is advised. Chest CT and routine cancer staging imaging may incidentally detect coronary calcification or intracardiac masses, aiding in the identification of subclinical CVD. Functional imaging such as stress echocardiography, perfusion CMR, or nuclear myocardial perfusion imaging should be considered in patients with ischemic symptoms, particularly before initiating therapies with vascular toxicity risk (e.g., fluoropyrimidines, VEGF inhibitors, BCR-ABL tyrosine kinase inhibitors). Coronary computed tomography angiography (CCTA) is a high-sensitivity alternative for patients with low to intermediate pre-test probability of coronary artery disease (CAD) [88]. Cardiopulmonary exercise testing (CPET) provides a comprehensive assessment of cardiovascular function by evaluating the body’s ability to deliver and utilize oxygen during exertion, reflecting overall cardiorespiratory fitness (CRF). Peak oxygen consumption (VO_2_ peak) and metabolic equivalents (METs) are key indicators. CRF derived from CPET is a strong, independent predictor of cardiovascular health, overall mortality, and risk classification. Despite its prognostic value, routine pre-treatment use in cancer patients is limited, primarily serving pre-operative risk assessment in lung, colon, and rectal cancer surgery. Its role in predicting cardiovascular events before cardiotoxic cancer therapies requires further study [89].

Genetic testing has identified approximately 40 genes and single nucleotide polymorphisms (SNPs) associated with anthracycline-related cardiac dysfunction. While most studies have focused on germline variants, emerging evidence suggests tumor somatic mutations may also contribute to CTR-CVT, particularly with immunotherapies. For example, in ICI-associated myocarditis, identical clonal T-cell populations were found in the heart, tumor, and skeletal muscle, with tumor RNA expressing cardiac-specific genes. Although genetic variants linked to cancer therapy-related CVD have been documented, routine genetic testing is not currently recommended. Future personalized genetic profiling may enhance prediction of individual cardiotoxicity risk [78]. Collectively, these diagnostic strategies provide a detailed understanding of baseline cardiovascular status, enabling early detection of dysfunction and guiding personalized interventions, which is essential for implementing effective therapeutic approaches to prevent or reverse cancer-related cardiac complications.

As we know, pediatric heart failure presents unique diagnostic challenges. Shaddy et al. [90] emphasized the importance of age-specific diagnostic criteria that account for distinct etiologies such as congenital heart disease and metabolic disorders, the conditions that differ substantially from those seen in adults. Thus, standardized pediatric diagnostic protocols are essential for facilitating early detection and intervention [90,91]. Despite considerable advances, key challenges remain in standardizing diagnostic tools across varied clinical settings and ensuring their accessibility. Variability in echocardiographic interpretation, coupled with the high cost and limited availability of cardiac MRI, impedes universal adoption. Moreover, effective integration of multimodal diagnostic approaches requires a coordinated, multidisciplinary strategy to optimize patient outcomes. Persistent discrepancies in diagnostic criteria across clinical guidelines and health systems underscore the need for international collaboration to harmonize diagnostic standards and promote equitable access to advanced diagnostics worldwide.

## 6. Clinical Trials to Target Cancer Chemotherapy Induced Cardiac Cachexia

Several clinical trials have been completed or in progress to evaluate the efficacy of various chemo-drugs on cardiac functions in patients with various types of cancers. Table 2 summaries the chemo-drugs tested or being tested in patients with cardiac dysfunction. 

## 7. Therapeutic Approaches

### 7.1. Individualized Treatment

The decision to initiate cardiovascular (CV) treatment whether pharmacological or device-based must account for multiple factors, including the severity of both cancer and CV symptoms, cancer prognosis, the necessity and alternatives to ongoing cancer therapies, potential adverse drug reactions and drug–drug interactions, as well as the patient’s preferences. In cases of cancer therapy-related cardiac dysfunction induced by anthracyclines or HER2-targeted therapy, management depends on symptom severity. Temporary interruption or discontinuation of chemotherapy may be warranted in symptomatic patients. Conversely, asymptomatic patients with a preserved LVEF (≥50%) but elevated troponin levels are typically managed with ACE inhibitors, ARBs, or beta-blockers while continuing chemotherapy. For patients requiring anthracycline therapy restoration, liposomal formulations may be considered to reduce cardiotoxicity [74]. In suspected immune checkpoint inhibitor (ICI)-associated myocarditis, temporary discontinuation of ICI therapy is advised until the diagnosis is confirmed or excluded. If non-invasive assessments such as cardiac imaging and biomarkers are inconclusive, endomyocardial biopsy should be performed. Confirmed cases require withholding ICI therapy, continuous ECG monitoring for arrhythmias, and early initiation of high-dose corticosteroids as first-line therapy. Second-line immunosuppressive agents should be considered for corticosteroid-refractory cases. Fulminant myocarditis warrants ICU-level care, intravenous methylprednisolone, and comprehensive cardiovascular management, including mechanical circulatory support when necessary [92,93].

Interestingly, cardiovascular complications occur in approximately 20% of adults receiving Chimeric Antigen Receptor T-cell (CAR-T) therapies, often severe and associated with high mortality. Most CV events are secondary to cytokine release syndrome (CRS) or immune effector cell-associated neurotoxicity syndrome (ICANS), with arrhythmias (77.6%) including QTc prolongation, ventricular arrhythmias, and atrial fibrillation (AF) being the most common, followed by HF (14.3%), and less frequently myocardial infarction or venous thromboembolism (0.5%). Diagnosis relies on resting and continuous ECG, transthoracic echocardiography (TTE), and cardiac biomarkers (cTn, NPs). Severe cases may require ICU admission due to malignant arrhythmias, circulatory collapse, or multiorgan failure. CRS severity generally correlates with cytokine levels, particularly IL-6, whereas CRP is less specific. Management integrates standard ESC-guided CV care with CRS-directed therapies such as tocilizumab and dexamethasone [94]. Takotsubo syndrome (TTS) is more prevalent among cancer patients and is associated with worse outcomes. Malignancy itself, certain cancer therapies (e.g., 5-FU, ICIs, VEGF inhibitors), and cancer-related stress are recognized triggers. Diagnosis follows standard TTS criteria, including ECG, TTE, cardiac biomarkers, and cardiac MRI. Invasive coronary angiography is generally required to exclude acute myocardial infarction, with coronary CT angiography (CCTA) as an alternative when angiography is contraindicated. Early imaging is essential as left ventricular dysfunction may be transient; but repeat assessments might confirm recovery. Management includes interruption of the causative drug, avoidance of QT-prolonging medications, and, in ICI-associated TTS with evidence of myocardial inflammation, consideration of intravenous steroids [95]. We opine that individualized management is critical in cardio-oncology due to the heterogeneity of mechanisms and severity of CV complications across cancer therapies. Treatment strategies should be tailored based on cancer type, baseline cardiac risk, clinical presentation, and patient preferences to optimize outcomes. Early detection and prompt, mechanism-guided intervention, supported by multidisciplinary collaboration between cardiologists and oncologists, are essential to minimize therapy interruptions while safeguarding patient safety.

### 7.2. Preclinical Models

In a preclinical model of ovarian cancer, Withaferin A, a steroidal lactone derived from *Withania somnifera* (Ashwagandha), was administered at doses of 2 mg/kg and 4 mg/kg. The treatment led to improved left ventricular mass, 90 to 95% preservation of systolic and partial diastolic function, and prevention of reductions in cardiomyocyte size and cardiac troponin I (TnI) levels. These benefits were associated with suppression of pro-inflammatory cytokines, including TNF-α and AngII, and a reduction in pathologic MHC isoform switching, supporting the potential of Withaferin A as a candidate therapeutic agent for preserving cardiac function in cancer-bearing hosts [96]. Karekar et al. [13] investigated the contribution of reactive oxygen species (ROS) in tumor-induced cardiac dysfunction using mouse and Drosophila models. They found that tumorigenesis induced systemic increases in ROS levels which impaired cardiac function. Antioxidant treatments were able to reverse these effects suggesting that antioxidant therapy could be a viable approach to counteracting cancer-induced cardiac dysfunction. Finally, Ausoni et al. [97] described how progressive cancer exacerbates cardiac dysfunction through the systemic release of pro-cachectic factors and inflammatory mediators. Their review emphasized the importance of early diagnostic criteria and proactive interventions while also calling for innovative therapies targeting the molecular mechanisms of cancer-induced cardiotoxicity. Collectively, these therapeutic strategies aim to reduce chemotherapy-related cardiotoxicity, prevent cardiac remodeling, and preserve myocardial function. While several pharmacological agents and interventional procedures have demonstrated efficacy in reducing the incidence and severity of HF and cardiac atrophy, further high-quality, controlled studies are necessary to confirm these findings and establish robust treatment guidelines. Additionally, the effective management of cancer-induced cardiac dysfunction demands a comprehensive, multidisciplinary approach, combining evidence-based pharmacological interventions, targeted interventional strategies, and lifestyle modifications. In this pursuit, continued translational research is crucial to refining existing therapies, standardizing diagnostic and therapeutic protocols, and ultimately improving the long-term cardiovascular outcomes and quality of life for cancer patients.

### 7.3. Role of Stem Cells in Cardiac Regeneration

There are data indicating that heart muscle cells can regenerate in lower vertebrates and during the early neonatal period in mammals; however, this possibility is not efficient during the postnatal phase of life [98,99]. Studies measuring carbon-14 (^14^C), which increased in the environment during the 1950s and 1960s due to nuclear bomb testing, established the cell cycle activity of cardiomyocytes at a rate of about 0.5–1% per year [100]. This corresponds with other studies in mice labeled with ^3^H-thymidine or ^15^N-thymidine that revealed circulation at a rate below 1% per year. Finally, research in humans using the mitotic marker phosphorylated histone H3 (pH3) established a rate of cardiomyocyte cycling at less than 1.5% per year [101,102]. Based on this low rate of endogenous regeneration, several cellular strategies to regenerate myocardium have been proposed. These include (i) renewing pre-existing mature cardiomyocytes by stimulating their dedifferentiation and expansion, (ii) transdifferentiating non-cardiomyocytes through gene modification strategies, and (iii) applying induced pluripotent stem cells derived from cardiomyocytes alone or in combination with scaffolds. The most ideal option for myocardium regeneration would be to employ cardiac stem cells. There are described potential cardiac stem cells based on expression of the c-kit receptor, Sca-1 antigen, Mesp-1, and the Kdr receptor [103]. These cells could be isolated from bone marrow (BM) or directly from cardiac tissue (e.g., heart atria). From a historical perspective, bone marrow-derived cells have been studied for the regeneration of myocardium. While there was evidence of clinical improvement, this was not related to the replacement of new cardiomyocytes, but as is currently acknowledged, due to the paracrine effects of the cell’s secretome (microvesicles and secreted bioactive mediators).

An important limitation in heart regeneration is that the remaining cardiomyocytes have a very limited ability to replace lost cells in the myocardium. This inability to regenerate damaged tissue leads to scarring, which compromises proper cardiac function and often culminates in HF. To address this challenge, two main strategies have been explored in recent years. The first aims to stimulate resident cardiac stem cells (CSCs) that survive after myocardial injury, while the second seeks to generate new cardiomyocytes from putative CSC populations [104]. Several types of cells have been isolated from the myocardium and proposed as potential CSCs. Among these, resident c-kit+ cells captured significant attention because a single c-kit+ CSC was reported to differentiate into multiple cardiac lineages including cardiomyocytes, endothelial cells, and smooth muscle cells both in vitro and in vivo [105]. However, subsequent studies employing rigorous dual recombinase-activated lineage tracing demonstrated that these cells do not meaningfully contribute to new myocardium, and more advanced technologies have not validated the existence of functional c-kit+ CSCs [106]. Sca-1+ cells were also suggested as candidate CSCs [107]; yet further investigation revealed that this population is heterogeneous and primarily enriched in endothelial progenitors rather than true CSCs. The beneficial effects observed with Sca-1+ cell transplantation appear to result mainly from angiogenesis and paracrine signaling rather than direct cardiomyocyte regeneration [108]. Another proposed CSC population, Abcg2+ cells, was found to differentiate into multiple cardiac lineages during embryogenesis only, with no significant contribution to adult myocardial regeneration [109,110]. More recently, Bmi1+ cells have been proposed as a potentially regenerative population [111]. These cells represent a subpopulation of Sca-1+ cells, suggesting a limited but possible capacity to generate new cardiomyocytes. In parallel, significant efforts are underway to reprogram cardiac fibroblasts into functional cardiomyocytes through overexpression of specific transcription factors, offering a promising avenue for future research, including the development of small molecule-based strategies [104]. Additionally, the use of embryonic stem cells or induced pluripotent stem cell (iPSC)-derived cardiac progenitors and cardiomyocytes remains an area of active investigation. At present, the regenerative potential of these approaches largely relies on paracrine effects rather than direct replacement of lost cardiomyocytes. Understanding the limited regenerative capacity of the adult heart underscores the need for targeted therapeutic strategies that not only prevent or mitigate cancer therapy-related cardiac injury but also preserve myocardial function, setting the stage for the development of novel interventions in cardio-oncology.

## 8. Conclusions

Cancer-induced cardiac dysfunction represents a complex and evolving clinical challenge at the intersection of oncology and cardiology. As cancer treatments have advanced and survival rates have improved, the unintended cardiovascular consequences of antineoplastic therapies have become increasingly evident. Cardiac dysfunction manifesting as HF, cardiac atrophy, arrhythmias, and vascular complications poses a significant threat to the quality of life and long-term survival of cancer patients and survivors. Our review synthesizes current understanding of the multifaceted mechanisms underlying cancer-induced cardiac injury, advances in diagnostic modalities, and emerging therapeutic strategies that offer hope for improved patient outcomes. At the mechanistic level, it is now recognized that cardiac dysfunction in cancer extends beyond the direct cardiotoxic effects of chemotherapeutic agents. Tumor-derived factors such as pro-inflammatory cytokines (e.g., TNF-α, IL-6), reactive oxygen species (ROS), neurohormonal mediators (e.g., Ang II), and metabolic stress, they all contribute to systemic alterations that promote cardiomyocyte atrophy, fibrosis, mitochondrial dysfunction, and maladaptive remodeling. These biological disruptions are often exacerbated by cancer cachexia and immune dysregulation leading to a progressive decline in cardiac function. Importantly, such effects are not exclusive to chemotherapy but also arise in response to targeted therapies and immune checkpoint inhibitors, hence highlighting the need for comprehensive cardiac monitoring across diverse cancer treatment regimens. In terms of diagnosis, the integration of clinical evaluation with advanced imaging and biomarker profiling is essential for the timely and accurate identification of cardiac dysfunction. As mentioned earlier, echocardiography remains a cornerstone diagnostic tool, with enhancements such as tissue Doppler imaging and strain analysis offering greater sensitivity in detecting subclinical myocardial injury. Cardiac MRI provides superior tissue characterization and volumetric analysis, especially in infiltrative or fibrotic conditions. Additionally, lung ultrasound and cardiopulmonary exercise testing (CPET) are gaining traction as adjunct modalities, while biomarkers such as BNP, NT-proBNP, and troponins serve as accessible and prognostically meaningful indicators of myocardial stress. Despite these advances, there remains a pressing need to standardize diagnostic algorithms, increase accessibility to high-resolution imaging, and tailor pediatric protocols for early intervention in younger cancer patients.

Therapeutically, the field is witnessing a transition from reactive to proactive strategies. Statins, ACE inhibitors, beta-blockers, and dexrazoxane have shown clinical utility in preventing or attenuating cardiac damage, particularly in patients undergoing anthracycline or trastuzumab treatment. Emerging agents like endothelin receptor blockers and antioxidants are under investigation for their ability to reverse pathological remodeling. Novel interventions, including the use of Withaferin A—a bioactive compound with anti-inflammatory and cardioprotective properties—demonstrate the potential to bridge the gap between cancer therapy and cardiac preservation. These interventions highlight a paradigm shift toward precision cardio-oncology, where treatment is guided by molecular signatures, risk stratification, and close interdisciplinary collaboration. Looking forward, future research must focus on deepening our understanding of the molecular interplay between cancer progression and cardiac dysfunction. There is a critical need for large-scale, multicenter clinical trials to validate promising preclinical therapies, develop personalized medicine approaches, and refine surveillance protocols for high-risk populations. Furthermore, efforts to decode the role of microvascular dysfunction, immune cell infiltration, and metabolic derangements in cardiac pathology will likely open new therapeutic frontiers. Advances in computational modeling, systems biology, multi-omics, machine learning and artificial intelligence (ML-AI) technologies offer powerful tools to accelerate discovery and translation [112]. In conclusion, the recognition of cancer-induced cardiac dysfunction as a distinct clinical entity has catalyzed a new era of collaborative research and clinical innovation. Addressing this challenge requires a multidimensional approach that combines vigilant screening, evidence-based therapeutics and a set of novel research initiatives. By fostering synergy between oncologists, cardiologists, researchers and policymakers, we can certainly optimize cardiac health without compromising oncologic outcomes. With continued investment in translational research and clinical care infrastructure, the field is well-positioned to mitigate the burden of cardiac disease in cancer patients and usher in a new standard of cardio-oncologic excellence.

## Figures and Tables

**Table 1:** Distinguishing cancer-induced (cachexia, organs dysfunction) and chemotherapy-induced dysfunction (CTRCD).

	Cancer-induced toxicities (cachexia, organ dysfunction).	Therapy-induced toxicities (cachexia and organ dysfunction).
Primary cause	Tumor-secreted factors, systemic inflammation, metabolic disruption.	Direct organ toxicity from chemotherapeutics, radiation, and immunotherapy.
Mechanism	Chronic inflammation, catabolic signaling, and muscle/fat wasting.	Oxidative stress, cellular damage, hormonal imbalance, and inflammation amplification.
Effects	Muscle waste (skeletal and cardiac), weight loss, anemia, and immune suppression.	LV dysfunction, reduced LVEF, heart failure, immune suppression, cognitive impairment.
Organ involvement	Multiple organs including skeletal muscle, heart, liver, and kidney.	Primarily heart (LV systolic function), and other organs via systemic toxicity.
Diagnosis/Monitoring	Clinical assessment, cachexia staging, and metabolic markers.	Echocardiography (LVEF, Global Longitudinal Strain. GLS), biomarkers, cardiac imaging.
Treatment Approaches	Nutritional support, and anti-inflammatory agents under study.	Cardio-protective drugs (ACE inhibitors, beta-blockers), early detection, and long-term monitoring.

**Table 2:** Selected Clinical Trials on Cardiac Dysfunction in Cancer Patients (ClinicalTrials.gov), accessed on 30 November 2024).

Trial Identifier	Title	Primary Outcome Measure	Status/Phase	Cohort
NCT06268535	Identification of Anticancer Drugs Associated with Cancer Therapy-related Cardiac Dysfunction: A Pharmaco vigilance Study	Disproportionality analysis of individual case safety reports linking heart failure or cardiac dysfunction with cancer therapies	Completed	Patients with heart failure and cancer therapy-related dysfunction
NCT05851053	Breast Cancer Long-term Outcomes on Cardiac Functioning: a Longitudinal Study	Incidence of left ventricular systolic dysfunction	Recruiting	Breast cancer survivors
NCT05803889	Characterization and Kinetic of Chemotherapy-induced Cardiovascular Toxicity in Breast Cancer	Assessment of myocardial deformation (rest and submaximal effort) to characterize systolic and diastolic dysfunction	Recruiting	Stage I–III breast cancer patients receiving EC + paclitaxel
NCT02086695	Early Detection of Broken Hearts in Cancer Patients Bevacizumab, Sunitinib and Heart Failure	Changes in tissue velocity imaging, myocardial strain and strain rate, twist/torsion, and diastolic function indices	Completed	Adults (18–90 years) with metastatic renal cell carcinoma or colorectal cancer
NCT01434134	Prevention of Cardiac Dysfunction During Adjuvant Breast Cancer Therapy	Change in left ventricular ejection fraction assessed via cardiac MRI	Completed	Women (18–70 years) receiving adjuvant chemotherapy
NCT03790943	Cardiac Dysfunction in Childhood Cancer Survivors	Left ventricular ejection fraction measured by conventional echocardiography	Recruiting	Cancer survivors diagnosed at age 0–20 in Switzerland
NCT01904331	Breast Cancer Long-term Outcome of Cardiac Dysfunction (BLOC)	Association between cardiac dysfunction and therapy type; comparison with matched controls	Completed	Breast cancer patients
NCT05930418	Cardiovascular Magnetic Resonance Prognosticators in Pediatric Oncology Patients with Sepsis	Feasibility and prognostic value of cardiac MRI in pediatric oncology patients with sepsis	Recruiting	Patients aged 9–25 undergoing cancer treatment
NCT01641562	Diagnosis and Prediction of Taxanes Induced Cardiac Dysfunction	Change in LVEF post-treatment with taxanes; cardiotoxicity defined as LVEF < 55% or >10% reduction from baseline	Completed	Breast cancer patients > 18 years
NCT02496260	Biomarkers and Cardiac MRI as Early Indicators of Cardiac Exposure Following Breast Radiotherapy	Subclinical cardiac abnormalities on MRI correlated with subsequent cardiac events	Completed	Breast cancer patients > 18 years receiving radiotherapy
NCT05806138	A Study of Vericiguat in People with Breast Cancer and Cancer Therapy-Related Cardiac Dysfunction	Change in cardiorespiratory fitness (VO_2_ peak)	Recruiting	Breast cancer patients > 18 years
NCT01554943	Late Cardiac Evaluation of the Three Arm Belgian Trial Involving Node-positive Early Breast Cancer Patients	Incidence of asymptomatic (LVEF < 50%, NYHA I) and symptomatic cardiac events	Completed	Breast cancer survivors (18–90 years) without recurrence
NCT02605512	Breast Cancer and Cardiotoxicity Induced by Radiotherapy: the BACCARAT Study	Number of patients with reduced myocardial function by echocardiography	Completed	Surgically treated breast cancer patients (50–70 years) receiving adjuvant radiotherapy
NCT02494453	Pilot Study of Biomarkers and Cardiac MRI as Early Indicators of Cardiac Exposure Following Breast Radiotherapy	Subclinical cardiac abnormalities on MRI correlated with cardiac events	Completed	Breast cancer patients > 18 years undergoing left-sided radiotherapy
NCT03297346	Early Detection of Cardiovascular Changes After Radiotherapy for Breast Cancer	Decreased myocardial function assessed by echocardiography	Completed	Female unilateral breast cancer patients (40–75 years)

## Data Availability

No new data were created or analyzed in this study.
